# Reduced spore germination explains sensitivity of reef-building algae to climate change stressors

**DOI:** 10.1371/journal.pone.0189122

**Published:** 2017-12-05

**Authors:** Alexandra Ordoñez, Emma V. Kennedy, Guillermo Diaz-Pulido

**Affiliations:** 1 Griffith School of Environment and Australian Rivers Institute–Coast & Estuaries, Nathan Campus, Griffith University, Brisbane, Queensland, Australia; 2 ARC Centre of Excellence for Coral Reef Studies, Townsville, Queensland, Australia; Academia Sinica, TAIWAN

## Abstract

Reduced seawater pH and changes in carbonate chemistry associated with ocean acidification (OA) decrease the recruitment of crustose coralline algae (CCAcf.), an important coral-reef builder. However, it is unclear whether the observed decline in recruitment is driven by impairment of spore germination, or post-settlement processes (e.g. space competition). To address this, we conducted an experiment using a dominant CCA, *Porolithon* cf. *onkodes* to test the independent and combined effects of OA, warming, and irradiance on its germination success and early development. Elevated CO_2_ negatively affected several processes of spore germination, including formation of the germination disc, initial growth, and germling survival. The magnitude of these effects varied depending on the levels of temperature and irradiance. For example, the combination of high CO_2_ and high temperature reduced formation of the germination disc, but this effect was independent of irradiance levels, while spore abnormalities increased under high CO_2_ and high temperature particularly in combination with low irradiance intensity. This study demonstrates that spore germination of CCA is impacted by the independent and interactive effects of OA, increasing seawater temperature and irradiance intensity. For the first time, this provides a mechanism for how the sensitivity of critical early life history processes to global change may drive declines of adult populations of key marine calcifiers.

## Introduction

The persistence of marine benthic populations depends on the success of reproduction and early life history processes, such as fecundity, spore/gamete release, germination, settlement, and recruitment [1–3]. Therefore, understanding how global climate change affects marine populations requires consideration of the ecology of early stage processes. Our study focuses on the process of algal germination, which in the broader sense is defined as the stage after attachment in which a germ starts to sprout, grow and develop [1, 4]. Following spore maturation within the reproductive structures, spores are released to the external environment where they attach to the substrate and germinate. Subsequent cell division occurs, a germination disk is formed, and in the case of calcifying red algae, calcification takes place further cementing the crusts to the substrate, completing the settlement process [5–7]. Germlings and propagules are the stages most susceptible to mortality in many marine organisms life history because they can be killed by extreme habitat conditions or overgrown by other organisms, creating bottlenecks for marine populations [1, 3, 8]. Despite the fundamental importance of early life stages to most marine organisms, there is a critical knowledge gap of the interactive effects of climate change stressors (ocean acidification and warming) with other environmental factors on the development of marine algae (e.g. [9]), particularly on tropical populations.

Crustose coralline algae (CCA) are a group of calcifying red algae that play several important roles in marine ecosystems. CCA are a major calcifying component of the marine benthos, contributing to the formation and growth of tropical and temperate reefs worldwide through deposition of calcium carbonate (CaCO_3_), which also helps cement and stabilize reef framework [10, 11]. Further, coralline algae build algal ridges and maerl beds providing habitat and food for a number of organisms [12, 13]. Importantly, they are a preferred substrate for the settlement of many invertebrate larvae, especially reef-building corals [14]. This ability makes tropical CCA critical facilitators of the recovery of coral reefs after disturbances [15–18]. However, CCA are sensitive to the impacts of human-induced ocean acidification and warming [19–22] and it is unclear whether CCA will continue to be able to maintain these ecological functions under future climate change.

Ocean acidification (OA) is a global problem caused by increased carbon dioxide (CO_2_) emissions to the atmosphere, altering seawater carbonate chemistry [23]. OA reduces net calcification rates and can cause skeletal dissolution in many calcifying organisms including corals [22, 24], molluscs, foraminifera [25, 26] and CCA [21, 22, 27]. The skeletons of CCA are mainly composed of high-magnesium (High-Mg) calcite, a carbonate mineral form more soluble than the aragonite of corals [28], making CCA among the calcifying organisms most sensitive to OA [22, 27, 29]. Although responses of CCA to OA appear to vary between species [30–33], most studies show negative impacts of OA on biological and physiological processes of the individuals (e.g. growth, photosynthesis and net calcification [22, 34, 35]), populations, and on community composition of CCA [17, 30, 36–39]. Importantly, laboratory and field experiments show a reduction in the abundance of recruitment of CCA in response to elevated CO_2_, and it has been proposed that space competition with fleshy seaweeds is a major driver of the observed recruitment failure [37, 39, 40]. An alternative hypothesis is that OA directly reduces germination rates of early life stages of CCA, which has a negative implication for recruitment success. However, it is unknown whether spore germination of reef-building CCA is sensitive to elevated pCO_2_ and how climate change stressors independently, and in interaction with other environmental factors, affect the supply-side ecology of CCA [41].

Studies on adult stages of CCA show that elevated temperature exacerbates the negative impacts of OA causing tissue bleaching and mortality in tropical and temperate CCA [22, 38, 42]. High irradiance in combination with elevated pCO_2_ also decrease photosynthetic pigments and calcification on the articulated coralline alga *Corallina* [42]. However, it is unknown whether the effects of climate change stressors differ between the early and adult life history stages of reef-building coralline algae. In this study we first ask the question whether spore germination and early settlement of the key reef-building alga *Porolithon* cf. *onkodes* is sensitive to climate changes stressors, and secondly we ask whether the effects of pCO_2_ on the CCA are exacerbated by the interactions with increased seawater temperature and irradiance. To address these questions we conducted manipulative (factorial) experiments with different pCO_2_, temperature and irradiance levels using a flow-through experimental system at Heron Island on the Great Barrier Reef, Australia.

## Materials and methods

### Algae collection and life cycle

To investigate the effects of ocean acidification, temperature and irradiance on the germination of the CCA *Porolithon* cf. *onkodes*, we conducted multifactorial (orthogonal) laboratory manipulations of the three factors at Heron Island Research Station (HIRS), Great Barrier Reef (GBR), Australia. *P*. cf. *onkodes* was chosen because it is one of the most important reef-building CCA species in tropical regions [43] and is common in shallow GBR reefs [44], especially around Heron Island [45]. The algal collection and experiments were conducted in February-March 2014 during the summer season, which is when *P*. cf. *onkodes* is more reproductive [46]. Fertile fragments (3 x 3 cm) of tetrasporophytic plants were collected from the shallow reef crest (ca 5 m depth at highest tide) at Harry’s Bommie (Heron Island, GBR, 23°.27ˈ.631ˈˈS, 151°.55 ˈ.798 ˈˈE) using hammer and chisel. Fragments were transported to HIRS, carefully cleaned of epiphytes or other organisms and rinsed with sterilized seawater before spore release was induced. Algal collections were conducted under permit G12/34877.1 granted by Great Barrier Reef Marine Park Authority.

*Porolithon* cf. *onkodes* was identified based on field and laboratory observations. Laboratory techniques included Scanning Electron Microscopy and histology. *P*. cf. *onkodes* (Heydrich) Foslie is an encrusting coralline alga, with a smooth surface and granular appearance due to the presence of numerous tightly packed mega cells in horizontal fields (trichocytes fields), reproductive structures are contained in small, flush or slightly raised unipored conceptacles, cells are connected by fusions and thallus organization is non-coaxial [45, 47, 48]. All these characteristics are typical of *P*. *onkodes* (Heydrich) Foslie [47]. We have further used the colour of specimens in the field to make sure we were collecting the same species. Our specimens were pink-orange in colour. Our field observations and molecular work (in progress) indicate that crusts’ colour (for specimens occurring in the same light environment) can assist in the selection of samples to be collected to ensure that individuals of the same species are used in the experiment. Until the taxonomy of the *Porolithon “onkodes”* group is not resolved (e.g. [49]), we prefer to use *P*. cf. *onkodes* for our experimental species.

Fertile plants used in the experiment corresponded to the tetrasporophytic phase, which is a phase of their diplohaplontic and isomorphic (“Polysiphonia type”) sexual life cycle [7]. This means that the tetrasporangial plants (or tetrasporophytes, diploid, 2n) are morphologically similar to the gametangial plants (gametophytes, haploid, n). There is a further microscopic stage that develops inside the reproductive structure of a fertilized female plant (called the carposporophyte stage, which produces carpospores). Tetrasporophytic plants (2n) produce tetraspores (n) that are released to the environment and develop into haploid gamete-producing plants (n), the gametophytes (female or male). Tetraspores were the subject of this investigation. Coralline algae also have an asexual life cycle in which the tetrasporophytic plants produce self-perpetuating bisporangia. However, bisporangia were not observed in our samples.

Tetrasporophytic plants were recognised in the laboratory by removing the roof of the reproductive structures (conceptacles) using a blade and checking for the presence of tetraspores (zonately arranged spores). Carpospores are not zonately divided and are much larger than tetraspores (individual tetraspores measure 15–20 um), and conceptacles of male and female gametophytes produce spermatangia and carpogonia respectively, reproductive structures that are very different morphologically and anatomically to the tetrasporophytic structures [50]. Two to three conceptacles from every fertile CCA used in the experiment were examined and tetraspores were always found; spores could be easily seen with a dissecting microscope due to their red plastids [6]. We did not observe bispores during this examination. Based on these observations, it is unlikely that different phases of the sexual life cycle of *P*. *cf*. *onkodes* were included in this experiment.

### Spore preparation for germination analyses

**Fig 1:**
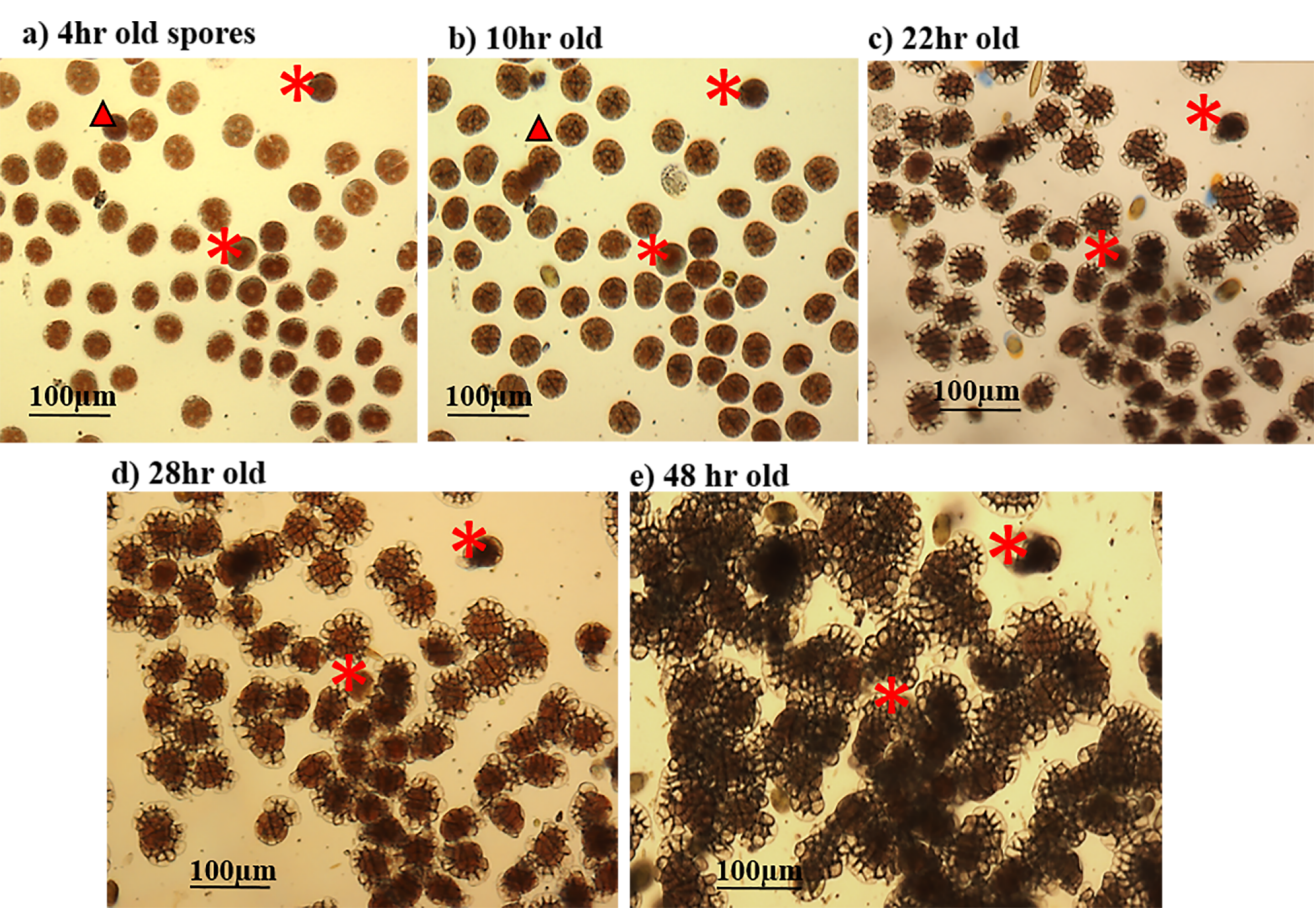
Sequential photographs of spore cell division, germling development and formation of germination disc in *Porolithon* cf. *onkodes*. Sequence shows spores after (a) four, (b) ten, (c) twenty-two, (d) twenty-eight, and (e) forty-four hours from spore release. Unsuccessful germination included those spores that neither initiate cell divisions nor formed a germination disc (indicating by *) and died or detached from petri dish (red triangle).

To obtain spores for the germination experiments we induced the release of spores following a combination of methods described in Jones and Moorjani [6], Ichiki et al. [51], Roleda et al.[52], however we did not quantify the actual release of spores or reproductive output of the experimental CCA. Fertile CCA were placed in a shallow tray without water in a dark cold room (18°C) for 30 min. Sterilized seawater was then added to the tray so that individuals were submerged (ca. 2 cm depth); individuals were then exposed to artificial halide and blue lights (160 μmol. photons m^-2^ s^–1^) for a period of 7 hours. After this period the majority of CCA began releasing tetraspores, and at this stage (i.e. onset of spore release), the adults with spores released on their surface were gently transferred to each experimental tank (1L plastic tanks) in which they were exposed to the experimental CO_2_, temperature and light conditions. Adults continue to release spores in the experimental tanks. We used three adult CCA fragments (belonging to different individual thalli) per experimental tank, to guarantee the availability of sufficient number of spores to commence the germination trials. Polystyrene, transparent petri dishes (94 mm × 16 mm diameter) positioned at the bottom of each experimental tank were used as a substrate for spore settlement and to facilitate manipulation when viewed under the microscope. Water flow produced by the incoming water into the experimental tanks (500 mL min–1) was sufficient to disperse spores on the petri dishes. After placing the adults in the tanks, 8 randomly selected petri dishes were examined every hour during 3–4 hrs for spore attachment and initial stages of cell division, indicative of the onset of germination. Attachment was recognised by the presence of a halo (mucus) surrounding the spore [6] and by gently brushing the spores to ensure spores did not detach easily. Commencement of the first cell division was initially detected in some spores at ca. 3–4 hours, and at this moment, adults were removed from the tank and a representative area on the petri dish was delimited and photographed using an Olympus digital camera DP72 mounted on a compound Microscope Olympus BX5 using a 10x objective lenses (the area of the microscope field analysed was 3.8mm^2^) for further analysis (this corresponded to T0 for our germination measurements, see below). Subsequent photographs of the same marked area were taken every 6 to 12 hrs for two days to study germination (Fig 1), and then every 24 hrs for additional two days for further population analyses. This photographic sequence allowed us to follow the development of individual spores through time. Adults were removed from the tanks to minimise a mixture of spores with different ontogenetic stages at the commencement of the measurements (see below), particularly since spore release under laboratory conditions is not synchronous among conceptacles (per. obs.). Despite removing the adults, the total number of spores varied across the experimental tanks at the beginning of the experiment (e.g. 10 to 60 spores were counted in microscopic fields of 3.8mm^2^).

### Experimental setup

Three replicate tanks, initially containing three individual adult thalli in each tank and later containing the spores of these adults were randomly allocated to each treatment combination of CO_2_ (three levels), temperature (two levels) and irradiance (two levels). There were a total of 12 treatment combinations and 36 experimental tanks. pCO_2_ and temperature were manipulated into 120 L mixing sumps (6 sumps: 3 CO_2_ levels x 2 temperature levels) and treated seawater was then continuously pumped into the experimental tanks at a rate of 500 mL min^–1^. The sumps were constantly fed with filtered seawater drawn from the Heron Island reef lagoon. Two types of filters were used: a hand-made sand filter containing a cotton filter mat and a house water filter OMNI OPAQUE with a 5 *μ*m cartridge to avoid contamination by spores from other algae coming from the reef lagoon. This way we excluded competing organisms such as filamentous algae during the experiment.

### pCO_2_ manipulations

pCO_2_ and temperature levels were chosen according to the RCP 8.5 model of the Intergovernmental Panel on Climate Change (IPCC) for CO_2_ stabilization scenarios [23] using standard protocols for OA research [53]. The RCP 8.5 model is the worst case scenario assuming constant increase of greenhouse gas emissions and continuous uptake of CO_2_ by the oceans which is projected to decrease pH by 0.30 to 0.32 by the end of the century (year 2100) [23]. The pCO_2_ levels included: Control (present day, no CO_2_ dosing, pCO_2_ ranged between 398 and 511 mg L^–1^ and pH NBS/NIST (pH National Bureau of Standards/National Institute of Standards and Technology) ranged from 7.81 to 8.01); medium (simulating levels predicted for 2050, target value: 540.5 mg L^–1^; calculated pCO_2_ ranged between 550.8 and 738.2 mg L^–1^, pH value from 7.82–7.91); and high (simulating levels predicted for 2100, target value 935.9 mg L^–1^; calculated pCO_2_ ranged between 998.9 and 1,568.8 mg L^–1^, pH value from 7.67–7.70) (S1 Table). pCO_2_ concentrations were manipulated by bubbling analytical grade CO_2_ into 120 L mixing sumps filled with filtered seawater. The aquarium control system (Aquatronica, AEB technologies, Italy) was used to monitor the seawater pH using temperature compensated pH electrodes (Mettler-Toledo, inPro4501VP), and temperature every 30 seconds within the mixing sumps. pH and temperature were also monitored in one experimental tank. When seawater pH in the mixing sumps exceeded the desired threshold, the control system opened solenoid valves to inject pCO_2_ into the mixing sumps as described in [54]. pH probes were calibrated daily and recalibrated with three NIST-certified pH buffers (Mettler-Toledo, Switzerland) to 0.01 pH units when necessary.

### Temperature manipulations

Two temperature levels were used: ambient control (25–27°C) and elevated temperature (27–29°C), representing two degrees above the average maximum summer temperature in the study area. Temperature was manipulated in the mixing sumps using aquarium heaters (Heater Jager 250W). In addition to monitoring pH and temperature using the Aquatronica system, both parameters were checked in twelve randomly selected experimental tanks every day for three days using a portable pH and temperature meter (Meter Toledo portable watertight IP67 dual-channel meter). This was to ensure pH and temperature constancy in experimental tanks compared to sumps during the experiment.

### Irradiance manipulations

Artificial halide lights (OCEANLIGHT PLUS 1X150W+2T5 24W) provided a 12h light: 12h dark photoperiod. Two irradiance treatment levels were used: high irradiance (140–160 *μ*mol photons m^-2^ s^–1^) and low irradiance (40–60 *μ*mol photons m^-2^ s^–1^); the latter achieved using a black mesh placed over experimental tanks. In Heron island, *P*. cf. *onkodes* can be found in a wide range of irradiances and depths in the fore-reef (Ringeltaube and Harvey [45] and per. obs.), from the intertidal to 8–10 m depth. We have recorded large variation in irradiance intensity in the fore-reef of the study site, from 800 *μ*mol photons m^-2^ s^–1^ in the intertidal to 50 *μ*mol photons m^-2^ s^–1^ at 8 m during sunny summer days. The irradiance levels used in our experiment mimic two light environments in which *P*. cf. *onkodes* can occur at a depth of 5–6 m: well-lit areas (140–160 *μ*mol photons m^-2^ s^–1^) and low irradiance microhabitats such as crevices (40–60 *μ*mol photons m^-2^ s^–1^)(per. obs.). All specimens used in the experiment were collected approximately at 6m from well-lit microhabitats. Light measurements in the reef and in the experiment were taken using the underwater quantum sensor LI-192 connected to the light meter LI-250A from LI-COR.

### Carbonate chemistry

Total alkalinity was measured for seawater sampled every six hours for 24 hours from the six sumps and one experimental tank of the CO_2_ x temperature treatment combination. One sample per sump, time and treatment combination was taken. Total alkalinity was measured using Gran titration with an open-cell potentiometric titrator (model T50 Mettler-Toledo). Subsamples (2–3) were run until a maximum 1% error was met. Results from sumps and tanks were compared to explore any potential contribution from algal metabolism to changes in carbonate chemistry, and no significant differences were found (Student’s t-test, p = 0.611, *n* = 18, see also S1 Fig for pH and temperature values). Therefore, values from both the sumps and tanks were averaged. Salinity was measured during the experiment using a portable refractometer and the average calculated was 34.5 (±0.5) mg L^–1^. Total alkalinity, pH and salinity values were used to estimate the concentration of dissolved inorganic carbon (pCO_2,_ HCO_3_^-^ and CO_3_^2-^) using Microsoft Excel CO_2_SYS version 2.1 [55]. The saturation state of seawater with respect to High-Mg calcite was calculated for a 16.4 mol% MgCO_3_, following protocol described in [20]. Carbonate chemistry parameters are shown in S1 Table.

### Response variables and data analyses

Germination was quantified using two complementary metrics: *germination success* and *germling growth rate*. *Spore germination success* was measured on 10 randomly selected spores from the microscopic field previously delimited on the petri dish by estimating the percentage of spores that successfully formed a germination disc. This estimate was recorded 48 (±3) hrs following the first observation of spore attachment (T0). Photographs taken between T0 and the final time (at 48 hr) were used to document spore development but not used in the quantification (Fig 1). Unsuccessful germination included spores that neither initiated cell divisions nor formed a germination disc. In addition, those spores that died or detached from the petri dish were included in this category (e.g. Fig 1). Because the number of spores varied considerably among microscopic fields (e.g. 10 to 60), and the process of germination occurring rapidly in our experimental species, we limited our observations to 10 spores in one microscopic field. *Germling growth rate* was estimated by the change in surface area of 5 individual germlings (spores) over a period of 48 hrs beginning at T0. A considerable number of spores coalesced during the course of the experiment (process not quantified in this study) and this limited the number of individual spores that could be followed effectively through time because the margin of the individual crusts fused with others.

**Fig 2:**
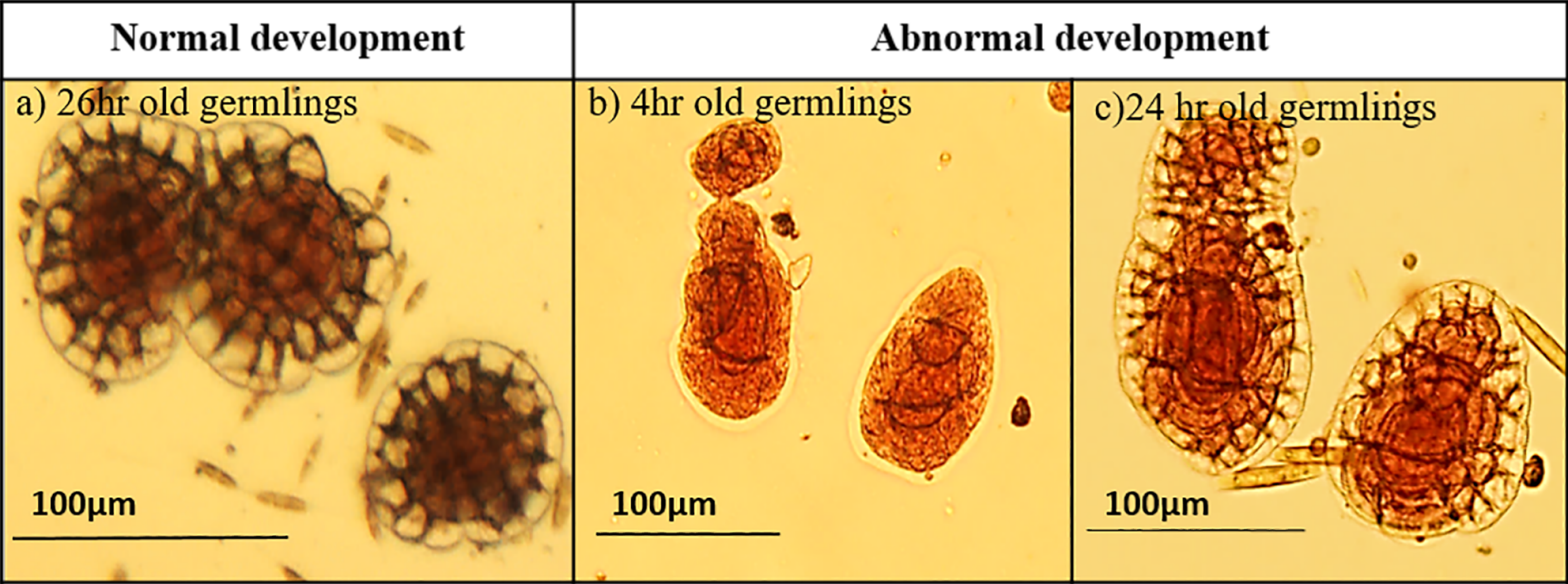
Normal and abnormal development of germlings of *Porolithon* cf. *onkodes*. (a) Normal germling development refers to when cell divisions and germination disc are clearly visible and development is circular and symmetrical. (b, c) Abnormal development shows irregular and enlarged cells, particularly in cells forming the germination disc.

In addition to germination metrics, we also quantified the *percentage of abnormalities* in germling development and this was assessed by calculating the proportion of germlings with abnormal cell divisions or sizes in a subsample of 10 spores. Normal development in this study refers to germlings whose cell divisions were clearly observed and followed a circular symmetry (especially during the first cell divisions) as described by Chihara [5] (see Fig 2A). Coralline algal spores follow a characteristic pattern of cell division to form a germination disc that is symmetrical and clearly visible even after few days of development (Chamberlain 1993). This characteristic pattern was considered “normal development” in this study. Finally, we calculated the *Change of percent area covered* by germlings: due to the coalescence of many germlings we were unable to continue measuring individual germling growth rate as the experiment progressed, therefore we additionally estimated the changes in total percent cover of the CCA population by measuring total area covered by all spores (coalescent and single spores) on the first day and fourth day of the experiment; data were normalized to initial cover to obtain a percentage. At the end of the experiment, photographs were analysed using Image J software (University of Wisconsin-Madison).

Response variables were analysed using a multifactorial, three-way ANOVA, with CO_2_ (three levels), temperature (two levels) and irradiance (two levels) as fixed factors and three experimental tanks as replicates. For germling growth rates, five germlings were measured per experimental tank, and values averaged per tank. When significant interactions occurred among factors, we conducted additional 2-way and one-way ANOVAs within treatment combinations [56], followed by post-hoc comparisons (Tukey test). Data normality and homogeneity of variance were tested using Kolmogorov-Smirnov and Cochran’s test respectively. Data were arc-sin transformed to meet criteria prior the analyses. Statistical analyses were performed using Systat 11.0.

## Results

### Germination success

**Fig 3:**
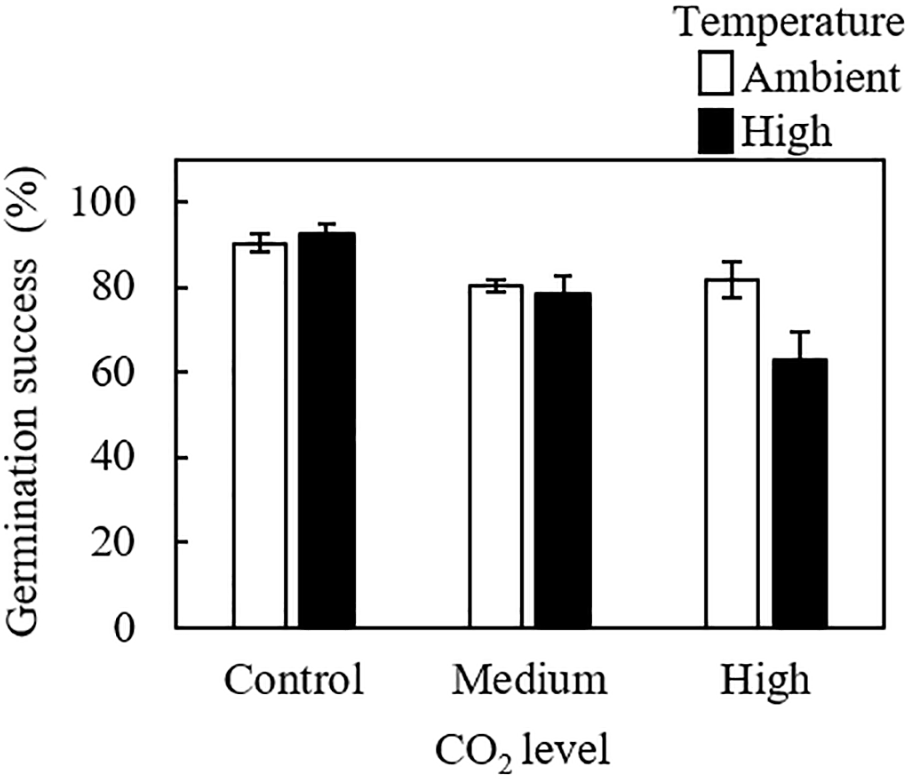
Effects of the combination of CO_2_ and temperature on spore germination success of *Porolithon* cf. *onkodes*. CO_2_ levels correspond to: control: 398–511 mg L^–1^, medium: 550–738 mg L^–1^ and high: 999–1568 mg L^–1^; temperature levels to: ambient: 25–27°C and high: 27–29°C. Letters above bars indicate significant groupings by post-hoc Tukey test. Since irradiance did not have an effect on spore germination success we pooled the data across irradiance levels, therefore data are means of n = 6 (± SE).

Elevated pCO_2_ reduced the percentage of spores that commenced germination compared to control pCO_2_ (Fig 3, S2 Table). However, there were significant interactions between CO_2_ and temperature on spore germination (three-way ANOVA, interaction term p = 0.032), showing that elevated temperature exacerbated the negative effect of CO_2_ on spore germination but only under high CO_2_. Under high CO_2_, germination was reduced from 81.7% in the ambient temperature to 62.7% under elevated temperature (ANOVA, p = 0.041, Fig 3, S2 Table), a decline of 23.2%. The irradiance treatment did not have any significant effect on spore germination (ANOVA, p = 0.9, S2 Table).

### Percentage of abnormalities in germling development

**Fig 4:**
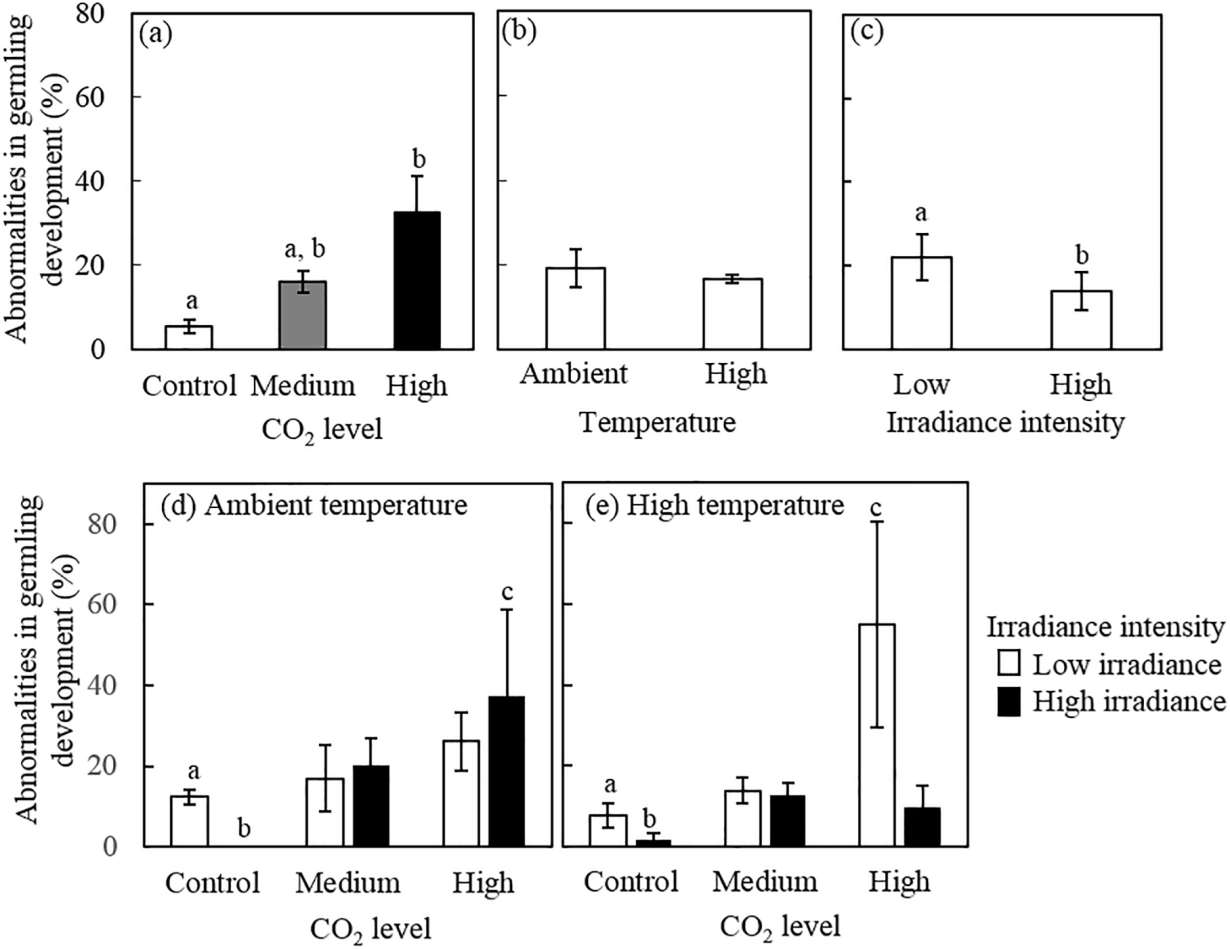
Effects of pCO_2,_ temperature and irradiance on the percentage of abnormal germlings of *Porolithon* cf. *onkodes*. (a), (b) and (c) show the main effects of pCO_2,_ temperature and irradiance respectively (means of n = 12 (± SE)). Lower panels show the individual effects of pCO_2_ and irradiance under ambient (d) and (e) high temperature (means of n = 3 (± SE)). CO_2_ levels correspond to: control: 398–511 mg L^–1^, medium: 550–738 mg L^–1^ and high: 999–1568 mg L^–1^; temperature levels to: ambient: 25–27°C and high: 27–29°C; and light levels to: high: 140–160 *μ*mol photons m^-2^ s –^1^ and low: 40–60 *μ*mol photons m^-2^ s –^1^. Letters above bars indicate significant groupings by post-hoc Tukey tests.

The proportion of abnormalities in germling development was also strongly affected by pCO_2_, with 33% of the spores showing abnormalities under high pCO_2_, compared to only 7% under control pCO_2_ (an 83% increase)(three-way ANOVA, p = 0.001, Fig 4A, S3 Table). Irradiance intensity negatively affected spore development with a higher percentage of abnormal spores observed at lower irradiance (21%) compared to higher irradiance intensity (13%, ANOVA, p = 0.042, Fig 4C, S3 Table). Temperature manipulations did not have a clear effect on the percentage of germling abnormalities (Fig 4B). Only marginal interactions among all the factors were found (ANOVA, p = 0.059, S3 Table), and this was explained by a significantly higher % of spore abnormality under the combination ambient CO_2_/ambient temperature in the low irradiance compared to high irradiance treatment (no abnormality was detected under the combination of control CO_2,_ ambient temperature and high light). Further, higher % of abnormality was also observed in the low irradiance treatment compared to the high irradiance treatment under the combination of control CO_2_/high temperature (p = 0.097) and high CO_2_/high temperature (p = 0.159), but the difference was not significant (Fig 4).

### Germling growth

**Fig 5:**
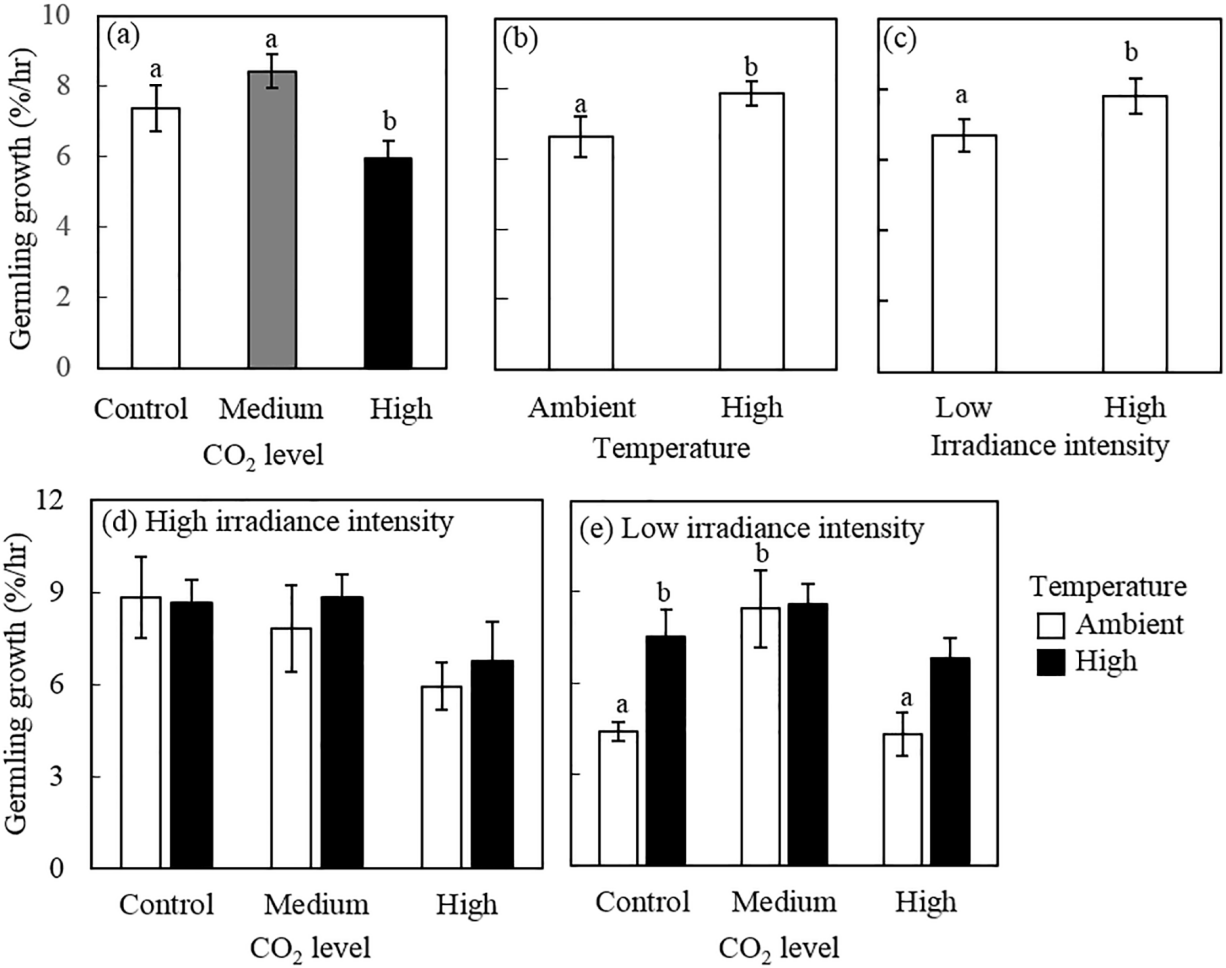
Effects of pCO_2,_ temperature and irradiance on germling growth rate (% change/hr) of *Porolithon* cf. *onkodes*. (a), (b) and (c) show the main effects of pCO_2,_ temperature and irradiance respectively (means of n = 12 (± SE)). Lower panels show the individual effects of pCO_2_ and temperature under high (d) and (e) low irradiance (means of n = 3 (± SE)). CO_2_ levels correspond to: control: 398–511 mg L^–1^, medium: 550–738 mg L^–1^ and high: 999–1568 mg L^–1^; temperature levels to: ambient: 25–27°C and high: 27–29°C; and light levels to: high: 140–160 *μ*mol. photons m^-2^ s –^1^ and low: 40–60 *μ*mol photons m^-2^ s –^1^. Letters above bars indicate significant groupings by post-hoc Tukey tests. Values represent growth rates from time of germination until 48 hours.

Germling growth rate was independently affected by pCO_2_, temperature and irradiance (ANOVA, p = 0.005, 0.035, 0.054, respectively, Fig 5A, 5B and 5C) and interactions between factors were not statistically significant (S4 Table). Growth rates showed a parabolic response to pCO_2_, with intermediate levels of CO_2_ inducing the highest growth rates (Fig 5A). We found an increase of 12.6% from control to medium pCO_2_ followed by a drop of 29.2% from medium to high pCO_2_ (Fig 5A). This response was evident only in the low irradiance treatment level, and more pronounced under ambient temperature (Fig 5E). Under high irradiance, there was a trend towards (although not significant) of decreased spore growth with increased pCO_2_ (Fig 5D).

Elevated temperature increased the rate of germling growth by 15.6% (main effect in the 3-way ANOVA, p = 0.035, Fig 5B). This increase was, however, more evident under low irradiance conditions, specifically in the control and high pCO_2_ levels (ANOVA, p = 0.029 and 0.067, respectively) (S4 Table). Germling growth rate increased by 70% from ambient to high temperature under control pCO_2,_ and by 57% under high pCO_2_ (Fig 5E).

The main effects of irradiance showed a reduction of germling growth rate from high irradiance intensity (7.8% h^-1^) to low irradiance intensity (6.5% h^-1^), a 14.3% drop (Fig 5C). This decrease was clearer under the combination of control CO_2_ and ambient temperature (Figs 5D and 4E, one-way ANOVA, p = 0.032) (S4 Table).

### Change of percent area covered by germlings

**Fig 6:**
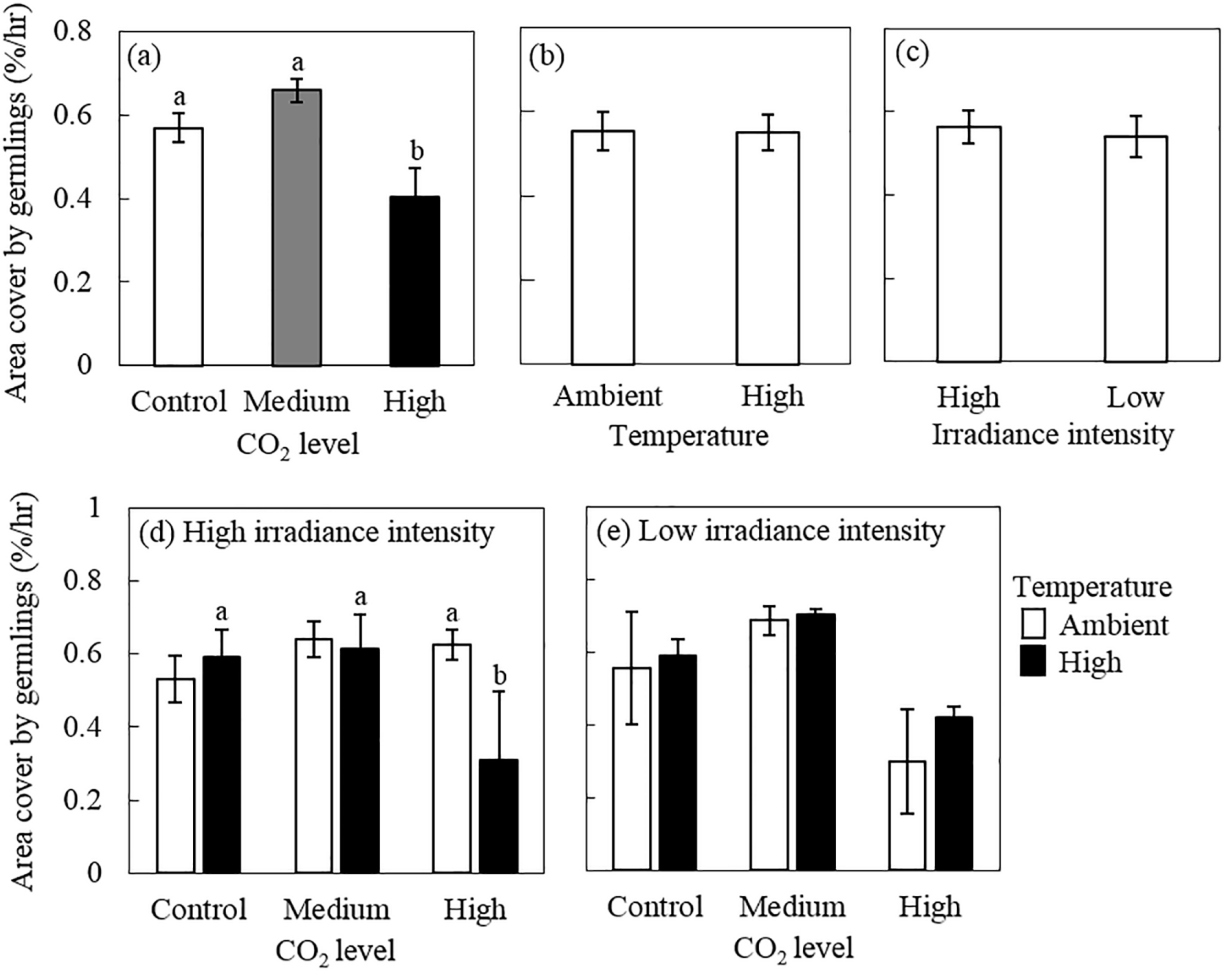
Effects of pCO_2_, temperature and irradiance on change in percentage cover of *Porolithon* cf. *onkodes* germlings over 5 days (change in %cover h-^1^). (a), (b) and (c) show the main effects of pCO_2_, temperature and irradiance respectively (means of n = 12 (± SE)). Lower panels show the individual effects of pCO_2_ and temperature under high (d) and (e) low irradiance (means of n = 3 (± SE)). CO_2_ levels correspond to: control: 398–511 mg L^–1^, medium: 550–738 mg L^–1^ and high: 999–1568 mg L^–1^; temperature levels to: ambient: 25–27°C and high: 27–29°C; and light levels to: high: 140–160 *μ*mol photons m^-2^ s –^1^ and low: 40–60 *μ*mol photons m^-2^ s –^1^. Letters above bars indicate significant groupings by post-hoc Tukey tests.

Similar to the rate of germling growth, the percent cover of juvenile CCA significantly increased in the medium pCO_2_ treatment level (ANOVA, p = 0.007, Fig 6A) (S5 Table). We found a decline in percent cover of germlings of 29% from control to high pCO_2_ and 39% from medium to high pCO_2_ (Fig 6A, S5 Table). When plotting the data by irradiance levels, this trend was consistent in the high and low irradiance intensity (Fig 6D and 6E). Neither temperature nor irradiance had an effect on total percent cover (Fig 6B and 6C).

## Discussion

Our study is the first to explore factor interactions in early life stages of CCA in the context of OA research and provides experimental evidence that increasing pCO_2_/low pH may affect germination of a major reef-building organism, both directly and in combination with other climate change and environmental factors. The effects of pCO_2_ on spore abnormalities and growth are stronger than the effects and interactions of the other factors we examined. This provides empirical support to a body of work that suggests a vulnerability of germlings and propagules of other groups of marine organisms to climate change (reviewed in [3]). As supply-side ecology processes are fundamental to marine populations [57], our results highlight the importance of studying not only adults but other stages of the life cycle to better understand the impacts of ocean acidification and warming on reef-building processes and reef resilience. CCA play critical roles in reef cementation and induction of coral larval settlement, therefore, a disruption of CCA germination and growth may impair reef stability and recovery potential as the oceans continue to acidify and warm in the future.

Importantly, we provide a mechanistic understanding of how recruitment failure occurs in CCA in response to OA. Elevated pCO_2_ reduces the rate of spore germination and growth, and produces abnormalities in recently germinated spores, independently of space competition with other organisms such as filamentous and fleshy algae, as suggested by earlier research [37, 39, 40, 58]. The formation of the germination disc and the development of spore cells, as well as the growth of germlings are all directly and negatively impacted by ocean acidification. Post-germination processes, such as the growth of germlings, however, increased with moderate pCO_2_ enrichment, suggesting that different aspects of the early life stages of CCA respond differently to environmental stressors, and that moderate CO_2_ increases may benefit growth of CCA juveniles. Our study isolated the effects of spore germination from space competition by filamentous algae as potential drivers of recruitment failure in CCA exposed to ocean acidification. However, filamentous algae can indirectly affect CCA spores, either positively (e.g. [59, 60]) or negatively (e.g. [37, 39, 40, 58]). Therefore, future studies should consider the relative contribution of space competition by filamentous algae and spore germination on CCA recruitment, preferably in natural habitats where the natural variability in ecological processes can be considered, variability that cannot be incorporated in laboratory experiments.

### Germination success

Spore germination in CCA is sensitive to elevated pCO_2_ and this sensibility is exacerbated considerably when spores are exposed to higher temperatures, such as those recorded during the austral summer in the southern GBR. We showed that spores incubated under the combination of elevated pCO_2_ and high temperature exhibited 40% less germination than spores kept under ambient pCO_2_ and temperature conditions. Although our study is the first to report inhibition of spore germination of coralline algae exposed to these conditions, other marine organisms also show similar responses, at least for elevated pCO_2_. For example, the unicellular green alga *Chlorella* experienced inhibition of cell division when exposed to CO_2_ oversaturation [61]. Multicellular spores of the giant kelp *Macrocystis* exposed to low pH (7.61) showed a significant reduction of spore germination [52]. The mechanisms explaining this decline are poorly understood, but could involve CO_2_ saturation in the boundary layer affecting the transport systems and gas exchange altering cell divisions [61], or an impairment in early calcification processes in CCA. The cellular processes by which elevated temperature exacerbates the negative response of spore germination to high pCO_2_ are unknown and require experimental examination. Synergistic effects of high pCO_2_ and high temperature have also been documented in adult CCA populations [22, 38], coral [62] and abalone larvae [63].

### Abnormalities in germling development

Abnormalities in CCA germlings were more frequent in spores exposed to high pCO_2_ than those growing under control conditions, suggesting a role of CO_2_ in altering the normal development of early stages of CCA (Fig 4A). A similar finding was reported for the Mediterranean CCA *Phymatolithon lenormandii* [41]. Cell enlargement, cell density reduction and cell wall thinning in adult CCA exposed to high pCO_2_ have also been described [64]. In our study, the abnormal germlings presented irregular and enlarged cells, especially among cells forming the germination disc (Fig 2). Irradiance variability also modified the normal development of germlings in our study, with more abnormal germlings occurring at low irradiance intensity agreeing with the fact that low irradiance does not seem optimal for *P*. *onkodes* development [43]. The reasons why low irradiance causes abnormalities is unclear, but it is likely that abnormal development is related to reduced availability of resources for growth (e.g. light). Spores from other macroalgae are quite sensitive to environmental stressors such as elevated nutrients [65] and high irradiance intensity [66]. The implications of spore abnormalities for subsequent early development, spore viability, and life cycle of CCA are unknown, but abnormal early development may impact adult populations as shown in terrestrial plants [e.g. *Arabidopsis* [67]].

### Germling growth and area cover by germlings

Growth rates of individual germlings and total cover of CCA germlings showed a parabolic response to elevated pCO_2_, with intermediate pCO_2_ enrichment favouring growth rates_._ This trend was clearer under low irradiance conditions (Fig 6E). This suggests that different response variables of the early life stages of CCA respond differently to environmental stressors. On the other hand, our results suggest that moderate pCO_2_ enrichment may in fact favour the growth of early stages of the CCA *P*. *onkodes*, as has been observed on some adult CCAs [19, 68]. CO_2_ is a substrate for photosynthesis, therefore elevated pCO_2_ can potentially alleviate CO_2_ limitation leading to enhancement of algal growth [e.g. as discussed in [69]]. This will depend on whether *P*. *onkodes* uses CO_2_ directly from the surrounding water via diffusion gradients, or whether it uses bicarbonate (HCO_3_^-^) as a source of carbon for photosynthesis [e.g. via carbon concentrating mechanisms (CCM)], as well as the ability to upregulate or down-regulate CCM activity [e.g. [70]]. Although we demonstrated that intermediate pCO_2_ concentrations favour the growth of early life stages of CCA, this only occurred under low irradiance conditions. Photosynthesis from non-calcifying red algae in low irradiance environments that lack CCM increases when exposed to high CO_2_ concentrations [71]. In these algae, CO_2_ is incorporated to the site of photosynthesis via direct diffusion gradients with little participation of CCM (e.g. [71–73]), and this may be a process by which CCA increase growth under moderate CO_2_ enrichment. Our results contrast with those from Bradassi *et al*. [41] who found that the growth of early stages of temperate CCA were not altered by variations in pCO_2_. Different methodologies were employed by Bradassi *et al*. [41]: they measured growth rates on two week old juveniles, while we recorded growth on 1–6 day old germlings making direct comparisons difficult. The decline in growth rates under elevated pCO_2_ is consistent with other studies that have shown declines in CCA recruitment with high pCO_2_ [27, 30, 39, 59]_._ We can infer that the decline observed in CCA recruitment under high pCO_2_/low pH in our experiment may be a direct result of the inability of the spores to commence cell division and germination disc formation and the decrease of germling growth rate.

Our study also demonstrates a positive effect of elevated temperature on individual germling growth rates. Elevated temperature in itself may enhance metabolic processes leading to increased algal growth which has been suggested for CCA [74] and other macroalgae [75]. The effects of temperature on CCA growth depends on species, temperature levels and interactions with other factors. For example, rising temperature accelerated the growth of germlings of a temperate coralline alga in Japan [51]. On the other hand, elevated temperature in combination with high pCO_2_ reduced CCA growth and calcification on tropical and temperate CCA, but temperature in itself did not have a significant effect on growth [21, 22]. Contrary to this, no interactive effects of pCO_2_ and temperature were observed on a tropical CCA [76]. Different results can also vary depending on the biological process being measured; for instance, diel net calcification in *Lithothamnion corallioides* increased under elevated CO_2_ with a progressive temperature increase from 10–19°C, while some photosynthetic pigments were reduced [32]. As discussed earlier, elevated temperature in our experiment did reduce germination (germination disc formation) but only under high pCO_2_. From these results and the literature we conclude that the effect of rising temperature can be positive or negative, but in general when temperature interacts with high pCO_2_ it synergistically impairs CCA physiology.

Variation in irradiance played a minor direct role in germling growth compared to the effects of pCO_2_ and temperature, however, irradiance interacted with the climate stressors to determine the direction and magnitude of the CCA responses. High irradiance slightly enhanced individual growth by 14%. This is not surprising as *P*. *onkodes* is generally found in well-lit shallow environments [43]. Few studies, however, have tested the interactive effects of irradiance with ocean acidification and warming on coralline algae. Comeau *et al*. [76] documented a decrease in net calcification of the CCA *Hydrolithon reinboldii* with rising pCO_2_ at low irradiance and ambient temperature, possibly as a consequence of photosynthesis reduction at low irradiance. As photosynthesis helps control the pH at site of calcification regulating the concentration of carbonate ion and consequently precipitation of CaCO_3_, a reduction of photosynthesis can impair calcification [76]. Growth rates of our experimental species dropped when exposed to the combined effects of low irradiance, elevated CO_2_ and ambient temperature, in line with Comeau *et al*. [76] findings. However, when temperature was increased, the direction of the response shifted enhancing algal growth rates, both under ambient CO_2_ and high CO_2_, alleviating the negative impacts of ocean acidification. As explained before, temperature may have accelerated physiological processes which could explain these results. Our study, once again, highlights the importance of considering multiple factors when studying the response to different environmental stressors.

Our study focussed on germination, an important process in the supply-side ecology of coralline algae. There are other early life history processes that may also be sensitive to the impacts of OA and that may exert considerable control on population and community structure of reef building CCA, but that have not been considered in the context of ocean acidification and climate change research. These include fecundity or reproductive output [65], spore release [1, 77], and spore coalescence [78]. Information on the direction and magnitude of the responses of these processes to environmental stressors will enable a better understanding of CCA dynamics. For instance, coalescence in seaweeds (a process observed but not quantified in our experimental CCA germlings) has been suggested to enhance growth on some fleshy macroalgae, potentially conferring adaptive advantages to populations of coalescing seaweeds [78].

## Conclusion

In conclusion, independently from other factors, elevated pCO_2_ has a strong negative effect directly on spore germination of the reef-building coralline alga *P*. cf. *onkodes*. It decreases spore germination success, generates abnormalities and hampers growth rates (although moderate pCO_2_ increase enhances growth). However, irradiance and temperature could intensify these effects. Our paper provides a mechanistic understanding of the likely causes of recruitment failure in CCA when exposed to OA, and identifies spore germination as a key process highly sensitive to the impacts of elevated pCO_2_. Subsequent competition for space with filamentous algae may exacerbate the negative effects of OA on recruitment (but see Short et al. [60]). Our results have also underlined the critical importance of simultaneously studying the effects of multiple stressors related to global environmental change, not only on the adult phase, but also on other stages of the life cycle of marine organisms. The influence of different environmental factors on the early stages of CCA will not only be reflected on adult populations and their communities, but on the role that these organisms play in coral reefs ecosystems.

## Supporting information

S1 Fig **Average (a) pH and (b) temperature measurements (**^**o**^**C) in experimental tanks and sumps across different CO**_**2**_ **and temperature treatment levels.** Data are means of n = 3 (± SD). Note that the experimental conditions in the sumps are the same as the experimental tanks. pH and temperature values were obtained using the aquarium control system Aquatronica, and the values from the tanks using a portable pH and temperature meter (Meter Toledo portable watertight IP67 dual-channel meter). Both probes were calibrated using the same NIST-certified buffers (Mettler-Toledo, Switzerland).
(DOCX)

S1 Table Summary of water chemistry parameters for the different CO_2_ and temperature levels. Data are means of n = 8 (± SE) (range).
(DOCX)

S2 Table Three way-ANOVA for the effects of CO_2_, temperature and irradiance on the percentage of *Porolithon* cf. *onkodes* spores that germinated. C = control CO_2;_ M = medium CO_2_; H = high CO_2_; AT = ambient temperature; HT = high temperature. MS = Mean square.
(DOCX)

S3 Table Three way-ANOVA for the effects of CO_2_, temperature and irradiance on the percentage of *Porolithon* cf. *onkodes* germlings with abnormal development. C = control CO_2;_ M = medium CO_2_; H = high CO_2_; HI = high irradiance; LI = low irradiance; AT = ambient temperature. MS = Mean square.
(DOCX)

S4 Table Three-way ANOVA for the effects of *p*CO_2_, temperature and irradiance on *Porolithon* cf. *onkodes* germling growth rate (%change in size/hour). C = control CO_2;_ M = medium CO_2_; H = high CO_2_; HL = high irradiance; LL = low irradiance; AT = ambient temperature; HT = high temperature. MS = Mean square.
(DOCX)

S5 Table Three way-ANOVA for the effects of CO_2_, temperature and irradiance on the total percentage cover of *Porolithon* cf. *onkodes* germlings. C = control CO_2,_ M = medium CO_2_, H = high CO_2_, HL = high irradiance, HT = high temperature. MS = Mean square.
(DOCX)
