# Supplementary material for: Reduced spore germination explains sensitivity of reef-building algae to climate change stressors
Source: PLoS One. 2017 Dec 5;12(12):e0189122. doi: 10.1371/journal.pone.0189122 (PMC5716602; doi:10.1371/journal.pone.0189122)
Supplement: S1 Fig — Average (a) pH and (b) temperature measurements (oC) in experimental tanks and sumps across different CO2 and temperature treatment levels. Data are means of n = 3 (± SD). Note that the experimental conditions in the sumps are the same as the experimental tanks. pH and temperature values were obtained using the aquarium control system Aquatronica, and the values from the tanks using a portable pH and temperature meter (Meter Toledo portable watertight IP67 dual-channel meter). Both probes were calibrated using the same NIST-certified buffers (Mettler-Toledo, Switzerland). (DOCX) [file pone.0189122.s001.docx]

**S1 Fig.** Average (a) pH and (b) temperature measurements (^o^C) in experimental tanks and sumps across different CO_2_ and temperature treatment levels. Data are means of n=3 (± SD). Note that the experimental conditions in the sumps are the same as the experimental tanks. pH and temperature values were obtained using the aquarium control system Aquatronica, and the values from the tanks using a portable pH and temperature meter (Meter Toledo portable watertight IP67 dual-channel meter). Both probes were calibrated using the same NIST-certified buffers (Mettler-Toledo, Switzerland).
