# Supplementary material for: Reduced spore germination explains sensitivity of reef-building algae to climate change stressors
Source: PLoS One. 2017 Dec 5;12(12):e0189122. doi: 10.1371/journal.pone.0189122 (PMC5716602; doi:10.1371/journal.pone.0189122)
Supplement: S1 Table — Data are means of n = 8 (± SE) (range). (DOCX) [file pone.0189122.s002.docx]

**S1** **Table.** Summary of water chemistry parameters for the different CO_2_ and temperature levels. Data are means of n=8 (± SEM) (range).

| **Treatment** | **Temperature**  **°C** | **TA**  **µmol kg^-1^** | **pH** | ***p*CO_2_**  **µatm** | **HCO^3-^**  **µmol kg^-1^** | **CO_3_^2-^**  **µmol kg^-1^** | **ΩHighMgCalcite 16.4mol%MgCO_3_** |
| --- | --- | --- | --- | --- | --- | --- | --- |
| **Control CO_2_**  **Ambient T** | 26.1  (±0.3)  (25.0-27.2) | 2323.7  (±4.8)  (2319.35-2333.61) | 8.01  (±0.005)  (8.0-8.03) | 425  (±6.4)  (400.8-448.1) | 1801.76  (±10.1)  (1763.1-1846.1) | 213.34  (±2.8)  (201.7-220.9) | 1.14  (±0.02)  (1.06-1.19) |
| **Control CO_2_**  **High T** | 27.83  (±0.2)  (27.3-28.9) | 2343.4  (±13.5)  (2290.83-2389.30) | 7.93  (±0.025)  (7.82-7.98) | 554.01  (±43.7)  (458-752) | 1870.52  (±31.1)  (1786.1-2007.7) | 193.82  (±8.1)  (157.1-209) | 1.06  (±0.05)  (0.85-1.17) |
| **Medium CO_2_**  **Ambient T** | 26.1  (±0.3)  (25.0-27.2) | 2337.0  (±13.4)  (2279.22-2377.81) | 7.86  (±0.012)  (783-7.91) | 646.61  (±19.5)  (580-709.2) | 1938.75  (±14.6)  (1912.6-2008.8) | 163.19  (±5.2)  (149.6-182.9) | 0.87  (±0.03)  (0.79-0.99) |
| **Medium CO_2_**  **High T** | 27.83  (±0.2)  (27.3-28.9) | 2343.1  (±10.4)  (2300.13-2377.52) | 7.88  (±0.015)  (7.82-7.92) | 617.12  (±28.5)  (738.2-550.8) | 1907.99  (±20.2)  (1849.3-1993.8) | 178.46  (±5.6)  (157.2-198.7) | 0.97  (±0.03)  (0.85-1.1) |
| **High CO_2_**  **Ambient T** | 26.1  (±0.3)  (25.0-27.2) | 2347.4  (±8.9)  (2311.55-2379.98) | 7.69  (±0.004)  (7.67-7.7) | 1026.67  (±9.4)  (99.89-1070.6) | 2065.06  (±7.6)  (2038-2096.9) | 115.95  (±1.4)  (110.1-120.6) | 0.61  (±0.01)  (0.58-0.65) |
| **High CO_2_**  **High T** | 27.83  (±0.2)  (27.3-28.9) | 2344.9  (±10.3)  (2301.8-2389.14) | 7.66  (±0.02)  (7.5-7.68) | 1129.97  (±68.5)  (1050.3-583.4) | 2064.59  (±16)  (2021.9-2176.5) | 115.23  (±4.6)  (86.9-124.2) | 0.61  (±0.03)  (0.47-0.69) |
