# Supplementary material for: Reduced spore germination explains sensitivity of reef-building algae to climate change stressors
Source: PLoS One. 2017 Dec 5;12(12):e0189122. doi: 10.1371/journal.pone.0189122 (PMC5716602; doi:10.1371/journal.pone.0189122)
Supplement: S2 Table — C = control CO2; M = medium CO2; H = high CO2; AT = ambient temperature; HT = high temperature. MS = Mean square. (DOCX) [file pone.0189122.s003.docx]

**S2 Table.** Three way-ANOVA for the effects of CO_2_, temperature and irradiance on the percentage of *Porolithon cf. onkodes* spores that germinated. C= control CO_2;_ M= medium CO_2_; H= high CO_2_; AT= ambient temperature; HT =high temperature. MS =Mean square.

| Source of variation | Df | MS | *F*-value | *P-*value | Conclusions  Tukey test |
| --- | --- | --- | --- | --- | --- |
| *Germination* |  |  |  |  |  |
| CO**_2_** | 2 | 171.21 | 3.631 | AT= 0.052 | C>M=H |
|  | 2 | 1344.59 | 9.352 | HT= 0.002 | C>M>H |
| Temp | 1 | 17.706 | 0.61 | C= 0.453 | n.s |
|  | 1 | 12.948 | 0.215 | M= 0.652 | n.s |
|  | 1 | 1086.36 | 5.507 | H= 0.041 | AT>HT |
| Irradiance | 1 | 1.568 | 0.016 | 0.900 | n.s |
| CO**_2_** * Temp | 2 | 384.083 | 3.983 | 0.032 | S |
| CO**_2_** * Irradiance | 2 | 157.392 | 1.632 | 0.217 | n.s |
| Temp * Irradiance | 1 | 17.791 | 0.184 | 0.671 | n.s |
| CO**_2_** * Temp * Irradiance | 2 | 107.508 | 1.115 | 0.344 | n.s |
| Error | 24 | 96.443 |  |  |  |
