# Supplementary material for: Reduced spore germination explains sensitivity of reef-building algae to climate change stressors
Source: PLoS One. 2017 Dec 5;12(12):e0189122. doi: 10.1371/journal.pone.0189122 (PMC5716602; doi:10.1371/journal.pone.0189122)
Supplement: S3 Table — C = control CO2; M = medium CO2; H = high CO2; HI = high irradiance; LI = low irradiance; AT = ambient temperature. MS = Mean square. (DOCX) [file pone.0189122.s004.docx]

**S3 Table.** Three way-ANOVA for the effects of CO_2_, temperature and irradiance on the percentage of *Porolithon cf. onkodes* germlings with abnormal development. C= control CO_2;_ M= medium CO_2_; H= high CO_2_; HI= high irradiance; LI= low irradiance; AT= ambient temperature. MS =Mean square.

| Source of variation | Df | MS | *F*-value | *P-*value | Conclusions  Tukey test |
| --- | --- | --- | --- | --- | --- |
| *Abnormal spores* |  |  |  |  |  |
| CO_2_ | 2 | 0.517 | 8.882 | 0.001 | C<H=M |
| Temp | 1 | 0.007 | 0.116 | 0.736 | n.s |
| Irradiance | 1 | 0.267 | 4.597 | 0.042 | LI>HI |
| CO_2_ * Temp | 2 | 0.003 | 0.048 | 0.953 | n.s |
| CO_2_ * Irradiance | 2 | 0.083 | 1.427 | 0.260 | n.s |
| Temp * Irradiance | 1 | 0.124 | 2.134 | 0.157 | n.s |
| CO_2_ * Temp * Irradiance | 2 | 0.186 | 3.201 | 0.059 | S |
| Error | 24 | 0.058 |  |  |  |
