# Supplementary material for: Reduced spore germination explains sensitivity of reef-building algae to climate change stressors
Source: PLoS One. 2017 Dec 5;12(12):e0189122. doi: 10.1371/journal.pone.0189122 (PMC5716602; doi:10.1371/journal.pone.0189122)
Supplement: S4 Table — C = control CO2; M = medium CO2; H = high CO2; HL = high irradiance; LL = low irradiance; AT = ambient temperature; HT = high temperature. MS = Mean square. (DOCX) [file pone.0189122.s005.docx]

**S4 Table.** Three-way ANOVA for the effects of *p*CO_2_, temperature and irradiance on *Porolithon cf. onkodes* germling growth rate (%change in size/hour). C= control CO_2;_ M= medium CO_2_; H= high CO_2_; HL= high irradiance; LL= low irradiance; AT= ambient temperature; HT =high temperature. MS =Mean square.

| Source of variation | Df | MS | *F*-value | *P-*value | Conclusions  Tukey test |
| --- | --- | --- | --- | --- | --- |
| *Spores growth* |  |  |  |  |  |
| CO_2_ | 2 | 18.327 | 6.679 | 0.005 | C=M>H |
| Temperature | 1 | 13.704 | 4.994 | 0.035 | HT>AT |
| Irradiance | 1 | 11.256 | 4.102 | 0.054 | HL>LL |
| CO_2_ *Temperature | 2 | .995 | .363 | 0.700 | n.s |
| CO_2_ * Irradiance | 2 | 6.880 | 2.507 | 0.103 | n.s |
| Temperature * Irradiance | 1 | 4.201 | 1.531 | 0.228 | n.s |
| CO_2_ * Temperature * Irradiance | 2 | 3.249 | 1.184 | 0.323 | n.s |
| Error | 24 | 2.744 |  |  |  |
