# Supplementary material for: Reduced spore germination explains sensitivity of reef-building algae to climate change stressors
Source: PLoS One. 2017 Dec 5;12(12):e0189122. doi: 10.1371/journal.pone.0189122 (PMC5716602; doi:10.1371/journal.pone.0189122)
Supplement: S5 Table — C = control CO2, M = medium CO2, H = high CO2, HL = high irradiance, HT = high temperature. MS = Mean square. (DOCX) [file pone.0189122.s006.docx]

**S5 Table.** Three way-ANOVA for the effects of CO_2_, temperature and irradiance on the total percentage cover of *Porolithon cf. onkodes* germlings. C=control CO_2,_ M= medium CO_2_, H= high CO_2_, HL= high irradiance, HT =high temperature. MS =Mean square.

| Source of variation | Df | MS | *F*-value | *P-*value | Conclusions  Tukey test |
| --- | --- | --- | --- | --- | --- |
| CO_2_ | 2 | 0.001 | 6.486 | 0.007 | M>C>H |
| Temperature | 1 | 1.028E-05 | 0.073 | 0.789 | n.s |
| Irradiance | 1 | 6.116E-06 | 0.044 | 0.837 | n.s |
| CO_2_ * Temperature | 2 | 5.813E-05 | 0.415 | 0.666 | n.s |
| CO_2_ * Irradiance | 2 | 9.409E-05 | 0.672 | 0.522 | n.s |
| Temperature * Irradiance | 1 | 0.000 | 2.554 | 0.126 | n.s |
| CO_2_ * Temperature * Irradiance | 2 | 0.000 | 2.143 | 0.145 | n.s |
| Error | 19 | 0.000 |  |  |  |
